# Fourteenth Annual ENBDC Workshop: Methods in Mammary Gland Biology and Breast Cancer

**DOI:** 10.1007/s10911-023-09549-7

**Published:** 2023-10-06

**Authors:** Silke Blair Chalmers, Tanne van der Wal, Silvia Fre, Jos Jonkers

**Affiliations:** 1 Department of Biomedicine, Aarhus University, Aarhus, Denmark; 2 Swammerdam Institute for Life Sciences, University of Amsterdam, Science Park 904, Amsterdam, The Netherlands; 3 Department of Genetics and Developmental Biology, Institut Curie, INSERM U934, CNRS UMR3215, Paris, France; 4 Division of Molecular Pathology, Oncode Institute, Netherlands Cancer Institute, Plesmanlaan 121, Amsterdam, The Netherlands

**Keywords:** Mammary gland biology, Breast cancer, Involution, Development, Non-traditional models, Tumour initiation, Immunotherapy, Mammary stroma, Cancer dormancy, 3D bioprinting

## Abstract

The fourteenth annual workshop of the European Network for Breast Development and Cancer (ENBDC) on *Methods in Mammary Gland Biology and Breast Cancer* was held on April 26th − 29th in Weggis, Switzerland. For the first time, early career researchers organised and took part in an additional ECR workshop on the 26th of April, which was received with great enthusiasm. The topics of the main workshop included mammary branching and morphogenesis, novel experimental systems (model organisms), systemic influences on tumour progression and the tumour microenvironment. Novel and recent findings were shared across excellent oral and poster presentations.

## Introduction

On April 26th -29th 2023, the European Network for Breast Development and Cancer (ENBDC) held the 14th workshop on *Methods in Mammary Gland Biology and Breast Cancer* in Weggis, Switzerland. The primary goal of this annual workshop is to bring together researchers in an informal setting to encourage in-depth and unvarnished discussions of novel technologies and discoveries [1, 2]. As spring was about to commence, sunshine and rain alternated the scene of the meeting venue, seminar-hotel Rigi, located on the shore of the beautiful Lake Lucerne with panoramic mountain views. The attendees, consisting of 60 scientists from 14 different countries, invited speakers and speakers selected from the many excellent abstracts, discussed newly published and unpublished work during four scientific sessions. Two poster sessions, comprising a total of 37 posters, provided plenty of opportunity for additional interaction and discussion.

## Meeting Report

### ECR Workshop

For the first time in its history, the ENBDC workshop was preceded by a one-day workshop for early career researchers (ECRs), similar to the Gordon Research Seminars that precede every Gordon Research Conference. The inaugural ECR workshop, organised by Silke Blair Chalmers (Aarhus University, Denmark) and Tanne van der Wal (University of Amsterdam, the Netherlands), provided an opportunity for learning and networking through scientific sessions, joint discussions of technologies and protocols, and social activities. The scientific sessions consisted of engaging presentations from 9 talented postdocs and PhD students, selected by the organisers: Patrycja Szybowska (Oslo University Hospital, Norway), Oliver Cottrell (University of Manchester, UK), Aida Aide Luna Perez (University of Sheffield, UK), Benjamin Davies (University of Cambridge, UK), Calvin Rodrigues (Institut Curie, France), Mathilde Folacci (Aarhus University, Denmark), Neve Prowting (University of Bristol, UK), Marleen Aarts (University of Amsterdam, the Netherlands), and Marika Caruso (VIB-KU Leuven, Belgium). The presentations were very well received with active interaction between peers and constructive discussion of methods and results. The ECRs also participated in a technical workshop to troubleshoot their work surrounding the chosen topics “imaging and image analysis” and “inducible gene expression”. The organisers of the ECR workshop are grateful to all the participants of this inaugural event and are hopeful it will become a mainstay of future ENBDC meetings.

### Main Workshop

The main meeting commenced with a keynote address by Christine Watson (Emeritus Professor of Cell and Cancer Biology, University of Cambridge/ Fellow Emerita, Newnham College, Cambridge). The address provided an opportunity for the ENBDC community to acknowledge and celebrate the tremendous contributions Watson – who recently retired – has made to the field of mammary gland biology. Watson provided a compelling overview of her career, commencing with work identifying and characterising the role of the KRAB zinc finger protein ZFP157 (also named ROMA), in pregnancy and lactation [3–6]. Loss of ROMA in luminal cells results in continued DNA replication but a loss of cell division, resulting in an increase in binucleated cells with enhanced markers of DNA damage. Interestingly, loss of ROMA rescues the lactation failure observed in mice with mammary-specific GATA3 deletion.

Next, Watson outlined work exploring the role of mammary stem cells in mammary gland development (7). Through conducting neutral, single cell-labelling studies in mouse mammary glands, Watson and her team demonstrated that the clonal progeny of mammary stem cells in this setting are exclusively lineage restricted and distributed sporadically in branching ducts or alveoli. Watson proposed to name these progenitor cells ‘enduring progenitors,’ as their unipotent nature prohibit them from being true mammary stem cells [8].

Switching to involution, Watson discussed the characterisation of the cell death pathways in post-lactation mammary gland regression [9–11]. Originally considered to be driven by apoptosis, the pioneering work of the Watson lab demonstrated that involution occurs independently of executioner caspases, through lysosome-mediated programmed cell death (lysoptosis). Mechanistically, involution initiates STAT3 signalling, resulting in upregulation of lysosomal enzymes cathepsins B and L and downregulation of the cathepsin inhibitor SERPINA3G/SPI2A. Furthermore, STAT3 regulates the formation of large, LAMP2-positive lysosomal vesicles, as well as trafficking to lysosomes. Finally, STAT3 activity switches cell function from secretion to uptake of milk fat globules, which also drives cell death.

In the final part of her keynote lecture, Watson discussed recent work exploring the immune environment of the mammary gland [12]. They found that leukocytes in the mammary gland can intercalate between ductal luminal and basal cells or mimic the morphology of alveolar basal cells. During involution, the immune cell repertoire fluctuates and is particularly distinct between involution days 3–6. Interestingly, tumour cells injected at involution day 3 grew faster than cells injected in nulliparous mice, while cells injected at involution day 6 grew slower. This work opens the way for future studies exploring the role of immune environment of the mammary gland in physiological and disease contexts.

### Session 1: Mammary Gland Development, Branching and Morphogenesis

The first scientific session on “Dimensions of development, branching and morphogenesis” was chaired by Walid Khaled (University of Cambridge, UK). Biophysicist Edouard Hannezo (Institute of Science and Technology Austria, Austria) opened the session presenting insightful work exploring self-organized branching morphogenesis. Although branching morphogenesis occurs ubiquitously in glandular epithelia, the rules governing this process are not fully understood [13]. To explore this, Hannezo utilised a framework of branching and annihilating random walks to develop a computational model of mammary gland morphogenesis which predicts the final mammary gland architecture at the end of puberty with a high degree of accuracy [14]. Using this model, he demonstrated that branching structures follow a simple rule of stochastic branching coupled with neutral competition for space. Next, Hannezo described work in stem cell lineage tracing within the mammary gland [15]. Unexpectedly, this study found that most cells at the branching tip, while heterogenous, have the same capacity to contribute to tip growth. In the final part of his talk, Hannezo discussed work exploring the role of local repulsion and external guidance on shaping branching structures, using data from zebrafish fin innervation and mouse ear lymphatic system to show how local guides must play a role in producing optimally tiled branching networks [16].

Next, Renée van Amerongen (University of Amsterdam, the Netherlands) presented her unpublished work on WNT4 regulation in the mammary gland, starting with the important question of tissue-specific expression patterns. Although WNT signalling is a well-known oncogenic pathway in particular in colorectal cancer, its precise role in breast cancer remains undetermined, even though the first *Wnt* gene was discovered as a mammary oncogene in mice. As small aberrations in WNT signalling might contribute to mammary tumorigenesis, van Amerongen decided to first clarify the tissue-specific expression of WNTs in the mammary gland, focussing on WNT4, which is known to be downstream of progesterone signalling [17]. In both mouse and human, a regulatory enhancer hub was discovered where one conserved enhancer, tentatively called CRS4, enables looping between the enhancer hub and the *Wnt4* promoter. Interestingly, CRS4 itself seems to function as a hub for other enhancers, including for progesterone receptor (PR) dependent enhancers. Moreover, luminal specific transcription factors, including GRHL2, were discovered as critical *Wnt4* regulators.

### Session 2: Novel Models and Methods

The second session, focussing on “Novel models and methods for mammary gland studies” was chaired by Colinda Scheele (VIB-KU Centre for Cancer Biology, Leuven, Belgium). The session was commenced with an insightful presentation from Kate Hughes (University of Cambridge, UK), who divided her time between providing an overview of the comparative physiology of non-traditional models and describing her research in mammary gland development in ruminants. While acknowledging the central role of laboratory rodents in mammary gland research, Hughes highlighted how non-traditional animal models may provide additional insights into mammary gland biology due to similarities in their biology and histo-anatomy [18]. In concluding this enlightening section, Hughes cautioned against extrapolating across species when interpreting the literature. Next, Hughes outlined her research characterising ovine mammary terminal duct lobular units [19]. Despite mammary gland function being of critical importance in livestock, little is known about the development of this organ in sheep. To address this, Hughes conducted a comparative study between younger and older lambs. Mammary epithelial proliferation, determined by Ki67 positivity, was higher in younger lambs and clustered at invading points within the gland. Older lambs had higher levels of intraepithelial T-lymphocytes; a cell type postulated to act in a negative regulatory manner within the mammary gland. Furthermore, Hughes identified stromal hotspots of Ki67 colocalised with immune cell aggregates that resembled tertiary lymphoid structures, a hitherto unrecognised component of the ovine mammary gland immune system.

Sandra Schöniger (Discovery Life Sciences Biomarker Services GmbH, Kassel, Germany) gave the final presentation of the session, focussed on the characterisation of mammary tumours in pet rabbits. Rabbits may represent a novel model to study breast cancer, as they develop spontaneous mammary tumours, have a life expectancy of 6–13 years and relatively low housing costs. Other supporting factors are very similar histological features between mammary glands in humans and rabbits, and that the size of some rabbits that can allow repeated biopsy collections, e.g., to check effectiveness of cancer therapies [20]. Predictive and prognostic biomarkers of human breast cancer can also by analysed in rabbit mammary tumours. Schöniger and coworkers found that mammary tumours in pet rabbits frequently have secretory activity, and most commonly are estrogen and progestogen receptor negative [21, 22]. Immunological characterisation by the determination of tumour infiltrating lymphocytes and immunostaining for the immunosuppressive enzyme IDO1 uncovered that the majority of rabbit mammary tumours have low immunogenicity [23]. Schöniger suggested that pet rabbits may provide useful models to study secretory carcinomas, as well as low immunogenic triple-negative breast cancers.

### Session 3: Cancer as a Systemic Disease

Session 3, “Systemic influences on tumour progression and therapy response” was chaired by Jos Jonkers (Netherlands Cancer Institute, the Netherlands) and kicked off by Emmanuelle Charafe-Jauffret (Cancer Research Center, Marseille, France), who provided an in-depth overview of her work on tumour initiation and breast cancer heterogeneity [24, 25]. Preneoplastic lesions are commonly identified during routine screening; however, there is an inadequate understanding of how and why cells progress from normal to preneoplastic to malignant. To explore this phenomenon, Charafe-Jauffret expressed oncogenes in mammary cells at various stages of differentiation. RAS expression induced DNA damage in mature luminal cells, but not in undifferentiated mammary stem cells. Mammary stem cells escaped DNA damage due to the expression of the transcription factor ZEB1 and the methionine sulfoxide reductase MSRB3. This study elegantly demonstrated that cellular plasticity differs across mammary cell populations. Next, Charafe-Jauffret described the recent work of her team characterising the role of X-inactive specific transcript (XIST) in breast cancer [26]. In normal conditions, XIST is responsible for X chromosome inactivation. Aberrant expression of XIST is found in some breast tumours, but the role of XIST in this disease had not been characterised. Charafe-Jauffret and collaborators found that XIST loss impairs differentiation in human mammary stem cells and accelerates tumorigenesis and aggressiveness in orthotopic transplants. Mechanistically, XIST loss causes bi-allelic re-expression of the mediator subunit MED14, making differentiation of human mammary stem cells less favourable. Collectively, XIST loss causes epigenetic priming that modifies cellular plasticity, redirects cell fate, and induces a different type of tumour development.

The second invited speaker was Olga Blomberg (de Visser lab, Netherlands Cancer Institute, Amsterdam, The Netherlands), who presented her work on the role of eosinophils in the response to immune checkpoint blockage (ICB) in breast cancer [27]. Although ICB is a promising new strategy for cancer therapy, the majority of patients are unresponsive to these treatments. Work investigating this phenomenon has focussed primarily on T cells, despite immune responses relying on the crosstalk between the adaptive and innate immune cells. To further investigate this hurdle, Blomberg conducted immune profiling of patient samples, and noted that patients who were responsive to ICB had elevated eosinophils in comparison to non-responsive patients. Using genetically engineered mouse models of breast cancer, Blomberg demonstrated that ICB treatment increased interleukin 5 (IL5) secretion by CD4^+^ T cells, which in turn increased eosinophil production in the bone marrow. Furthermore, mice treated with ICB and cisplatin had improved responses in comparison to ICB treatment alone, which Blomberg discovered was due to increased IL33 levels promoting intra-tumoral eosinophil infiltration and subsequent activation of CD8^+^ T cells. Collectively, this study provided evidence of an IL5-IL33-eosinophil axis which promotes ICB response, and opens the possibility of future approaches utilising this axis to improve patient outcomes.

The last invited speaker in this session was Walid Khaled (University of Cambridge, UK), who graciously replaced a last-minute apology to present recent work exploring tumour initiation via single-cell analyses in human samples [28]. Understanding the early stages of tumour formation will undoubtably lead to better predictive models and preventive strategies; however, a comprehensive databank of human breast single-cell RNA expression profiles to facilitate such studies is currently missing. To address this, Khaled conducted single-cell RNA sequencing on human tissues from 55 donors with varying risk factors for breast cancer and compiled the first human breast cell atlas. Using this atlas, Khaled identified changes in the epithelial, stromal, and immune components which aligned with the natural progression of the tissue. Stratifying by cancer risk factors, such as parity, age, and germline mutations allowed the investigators to uncover alterations in the homeostatic cellular states of the tissue, which broadly were not restricted to changes in a single cell type. Unexpectedly, the immune component in *BRCA1/2* mutation carriers had gene signatures indicative of immune exhaustion, which may suggest immune escape mechanisms are present in very early stages of tumour formation. Furthermore, there were distinct differences in *BRCA1* carriers in comparison to *BRCA2* carriers. This atlas, which has already provided novel insights into cellular heterogeneity, will be a tremendous resource for the community.

The session then progressed to the first selected short talk for the meeting from Zuzana Sumbalova Koledova (Masaryk University, Brno, Czechia), who discussed recent work exploring the stromal environment during mammary gland morphogenesis via spatial transcriptome mapping. Although the stromal environment has been long recognised to play a key role in mammary gland branching morphogenesis, its molecular function and the potential existence of specialised stromal cells is still unknown. To address this, Sumbalova Koledova and her team conducted single-cell RNA sequencing on pubertal mammary stroma combined with spatial transcriptome mapping. Using this approach, they identified specialised, spatiotemporally restricted fibroblast subpopulations that were unique to pubertal mammary glands and coincided with the presence of terminal end buds. Extending this work by lineage tracing as well as in vitro studies in organoid co-cultures, they demonstrated the functional heterogeneity of fibroblast subpopulations during mammary morphogenesis. This work provides an extensive functional classification of fibroblasts roles in the developing pubertal mammary gland and will have implications for the understanding of our stromal involvement in other settings, including pathologies such as fibrosis or cancer.

The final short talk of the session was provided by Marta Ciwinska (VIB-KU Leuven Center for Cancer Biology, Belgium), who presented her work exploring alterations in mammary gland morphology upon oncogenic expression. Initial stages of breast cancer development, including how mutant cells spread and proliferate, and the responses of healthy tissue to mutant cells, are poorly understood. To explore this, Ciwinska utilised an inducible multi-colour reporter mouse model that recombined to stochastically express either a fluorescent reporter and an oncogene (KRAS^G12D^-RFP or PIK3CA^H1047R^-RFP) marking mutant cells, or fluorescent reporter only (GFP, YFP, or CFP) marking wild-type cells. Combining this model with lineage tracing and together with imaging, Ciwinska found that oncogenic expression in the mammary impacted the growth and survival of both, oncogene-expressing mutant cells and surrounding wild-type cells. She demonstrated that the impact on wild-type cells was cell lineage dependent, with wild-type basal and luminal cells responding differently to the presence of surrounding red labelled mutant cells. Moreover, the behaviour of mutant cells was found to differ depending on the oncogene expressed. This work provides novel insights into the dramatic morphological remodelling in the mammary gland following the emergence of pre-cancerous mutant cells.

### Session 4: Tumour Microenvironment

Momo Bentires-Alj (University of Basel, DBM, Basel, Switzerland) chaired the fourth and final session of the meeting, focussed on “The mammary microenvironment and tumour progression”. The session was commenced by Ilaria Malanchi (Francis Crick Institute, London, UK), who presented her recent work on metastatic dormancy and reactivation. In patients, disseminated tumor cells (DTCs) are often maintained in the tissue in a state of dormancy for many years prior to outgrowth. What causes DTC reactivation is largely unknown. Malanchi presented an in vivo model of dormancy-permissive tissue where metastatic cells are largely kept dormant to investigate what perturbations cause metastatic reactivation.

Clare Isacke (The Institute of Cancer Research, London, UK) gave an excellent overview of her work on the tumour microenvironment in estrogen receptor-positive (ER+) breast cancer [29]. Patients with ER + breast cancer are specifically prone to metastatic relapse for a long time following initial treatment due to dormant disseminated tumour cells. So far, the lack of mouse models of ER + breast cancer with a representable age has made it difficult to study the triggers of reawakening of these dormant cells. Isacke showed that DTCs from ER + breast cancer display a dormant phenotype in mice of younger age, but form lung metastasis in mice over 1 year of age. Upregulated expression of platelet-derived growth factor C (PDGFC) expression in the aged microenvironment and bleomycin-induced fibrosis can promote metastasis, showing the importance of the microenvironment in DTC activation. Blocking the receptor PDGFRA via treatment with imatinib was shown to suppress metastatic outgrowth of ER + DTCs, providing a promising treatment opportunity to prevent ER + relapses.

The first short talk of this session was provided by Amelia Hasenauer (ETH, Zurich, Switzerland), presenting her ground-breaking engineering work on 3D bioprinting of mammary gland tissue using decellularized matrix materials. She presented her workflow of creating biomaterial scaffolds by decellularization of the bovine mammary gland, producing material with a gelling temperature below 37 °C. By mixing this matrix with gelatine and a photo crosslinker she was able to produce a photoreactive biomaterial to use for 3D bioprinting, allowing flexibility in creating mammary gland-like shapes and extrusions. She showed adhesion of both MCF10A cells and primary bovine mammary epithelial cells to 3D printed biomaterials in a variety of shapes.

The final short talk of the meeting was presented by Qiang Lan (Institute of Biotechnology, University of Helsinki, Finland), who discussed his pioneering work on epithelial-mesenchymal interactions during embryonic mammary gland development, focussing on the initial branching events of the embryonic mammary gland epithelium [30]. Lan showed, by performing tissue recombinations with micro-dissected embryonic mammary and salivary glands, that initial branching morphogenesis is instructed by the stroma of the tissue of origin. However, the mode of branching was mammary epithelium dependent. Moreover, transcriptomic profiling and functional studies showed that mesenchymal WNT/β-catenin signalling, especially the downstream target IGF1, are critical regulators of mammary gland development.

## Awards and Conclusions

The DeOme Award for the best short presentation was given to Zuzana Sumbalova Koledova (Masaryk University, Brno, Czechia). In another first for the meeting, the poster prizes were renamed to pay tribute to researchers who have contributed tremendously both to their respective fields, as well and to ENBDC community. The Glukhova-Medina poster prize, named after Marina Glukhova and Dan Medina, was awarded to Oliver Cottrell (University of Manchester, UK) for his work on the oscillatory HES1 expression in MCF7 cell cycle progression and arrest. Calvin Rodrigues (Institut Curie, France), was awarded the Hynes-Streuli poster prize, which honours Nancy Hynes and Charles Streuli, for his work on the transcriptional responses induced by Notch activation in vivo in mouse basal mammary cells. Finally, the Watson-Rosen poster prize, celebrating Christine Watson and Jeffrey Rosen, was awarded to Larissa Mourao (VIB-KU Leuven Centre for Cancer Biology, Belgium), for her work on developing 3D human breast organoids to explore step-wise developmental stages during tumour initiation.

The broad range of topics and exciting new research presented during the 14th ENBDC meeting was met with great enthusiasm from all participants. It was decided unanimously that the ECR workshop was a success and would be held again. The 15th ENBDC will be held on May 1st – 4th 2024 and will include an ECR workshop on May 1st, to which all interested early career researchers in the field are most welcome and encouraged to attend. The meeting will be chaired by Silvia Fre (Institut Curie, Paris, France), and co-chaired by Walid Khaled (University of Cambridge, UK). The postdoc and PhD chairs will be Beata Kaczyńska (University of Helsinki, Finland), Maria Rafaeva (University of Basel, DBM, Basel, Switzerland) and Jaime Redondo (CNIO, Spain).
